# External validation of a triage tool for predicting cardiac arrest in the emergency department

**DOI:** 10.1038/s41598-022-12781-6

**Published:** 2022-05-24

**Authors:** Jen-Tang Sun, Chih-Chun Chang, Tsung-Chien Lu, Jasper Chia-Cheng Lin, Chih-Hung Wang, Cheng-Chung Fang, Chien-Hua Huang, Wen-Jone Chen, Chu-Lin Tsai

**Affiliations:** 1 Department of Emergency Medicine, Far Eastern Memorial Hospital, New Taipei, Taiwan; 2 School of Medicine, College of Medicine, Tzu Chi University, Hualien, Taiwan; 3 Department of Clinical Pathology, Far Eastern Memorial Hospital, New Taipei, Taiwan; 4 Department of Nursing, Cardinal Tien Junior College of Healthcare and Management, Yilan, Taiwan; 5 Department of Emergency Medicine, National Taiwan University Hospital, 7 Zhongshan S. Rd, Taipei, 100 Taiwan; 6 Department of Emergency Medicine, College of Medicine, National Taiwan University, Taipei, Taiwan

**Keywords:** Medical research, Signs and symptoms

## Abstract

Early recognition and prevention comprise the first ring of the Chain of Survival for in-hospital cardiac arrest (IHCA). We previously developed and internally validated an emergency department (ED) triage tool, Emergency Department In-hospital Cardiac Arrest Score (EDICAS), for predicting ED-based IHCA. We aimed to externally validate this novel tool in another ED population. This retrospective cohort study used electronic clinical warehouse data from a tertiary medical center with approximately 130,000 ED visits per year. We retrieved data from 268,208 ED visits over a 2-year period. We selected one ED visit per person and excluded out-of-hospital cardiac arrest or children. Patient demographics and computerized triage information were retrieved, and the EDICAS was calculated to predict the ED-based IHCA. A total of 145,557 adult ED patients were included. Of them, 240 (0.16%) developed IHCA. The EDICAS showed excellent discrimination with an area under the receiver operating characteristic (AUROC) of 0.88. The AUROC of the EDICAS outperformed those of other early warning scores (0.80 for Modified Early Warning Score [MEWS] and 0.83 for Rapid Emergency Medicine Score [REMS]) in the same ED population. An EDICAS of 6 or above (i.e., high-risk patients) corresponded to a sensitivity of 33%, a specificity of 97%, and a positive likelihood ratio of 12.2. In conclusion, we externally validated a tool for predicting imminent IHCA in the ED and demonstrated its superior performance over other early warning scores. The real-world impact of the EDICAS warning system with appropriate interventions would require a future prospective study.

## Introduction

In-hospital cardiac arrest (IHCA) remains a major public health problem worldwide, with an estimated 290,000 IHCA events per year in the United States^1–4^. About 10% of IHCAs occur in the emergency department (ED)^2^, where infrequent physiologic measurements, ED crowding, and unstable patient conditions all contribute to this devastating outcome^2,5–7^.

Early recognition and prevention comprise the first ring of the Chain of Survival for in-hospital cardiac arrest (IHCA)^8^. The use of early warning scores (EWSs) has the potential to achieve this goal and has been extensively studied in inpatient unitsx^9–12^. Few studies have attempted to validate ward-based IHCA prediction tools in the ED with variable performance^13–15^. A previous systemic review indicated that several EWSs had an excellent predictive ability for 2-day in-hospital mortality (with or without resuscitation) after admission from the ED, but none of the EWSs adequately predicted clinical deterioration or resuscitated cardiac arrest in the ED^16^. As such, we previously developed and internally validated an emergency department (ED) triage tool, Emergency Department In-hospital Cardiac Arrest Score (EDICAS), for predicting ED-based IHCA^17,18^.

The EDICAS comprises an 8-item composite score with a possible range of 0 to 13, including age, arrival mode, and categorized vital signs with simple cutoff points trained by the ED data. In our previous study, patients with EDICAS of 6 or above may have a higher risk of developing IHCA in the ED, and higher levels of monitoring are suggested if available. In the original study, that EDICAS served as an excellent discriminator of ED-based IHCA, with an area under the receiver operating characteristic (AUROC) of 0.86 in the internal validation set. However, the predictive performance of EDICAS in external validation remains unclear. Therefore, we aimed to externally validate this novel tool in another ED population of a tertiary medical center.

## Methods

### Study design and setting

**Figure 1:**
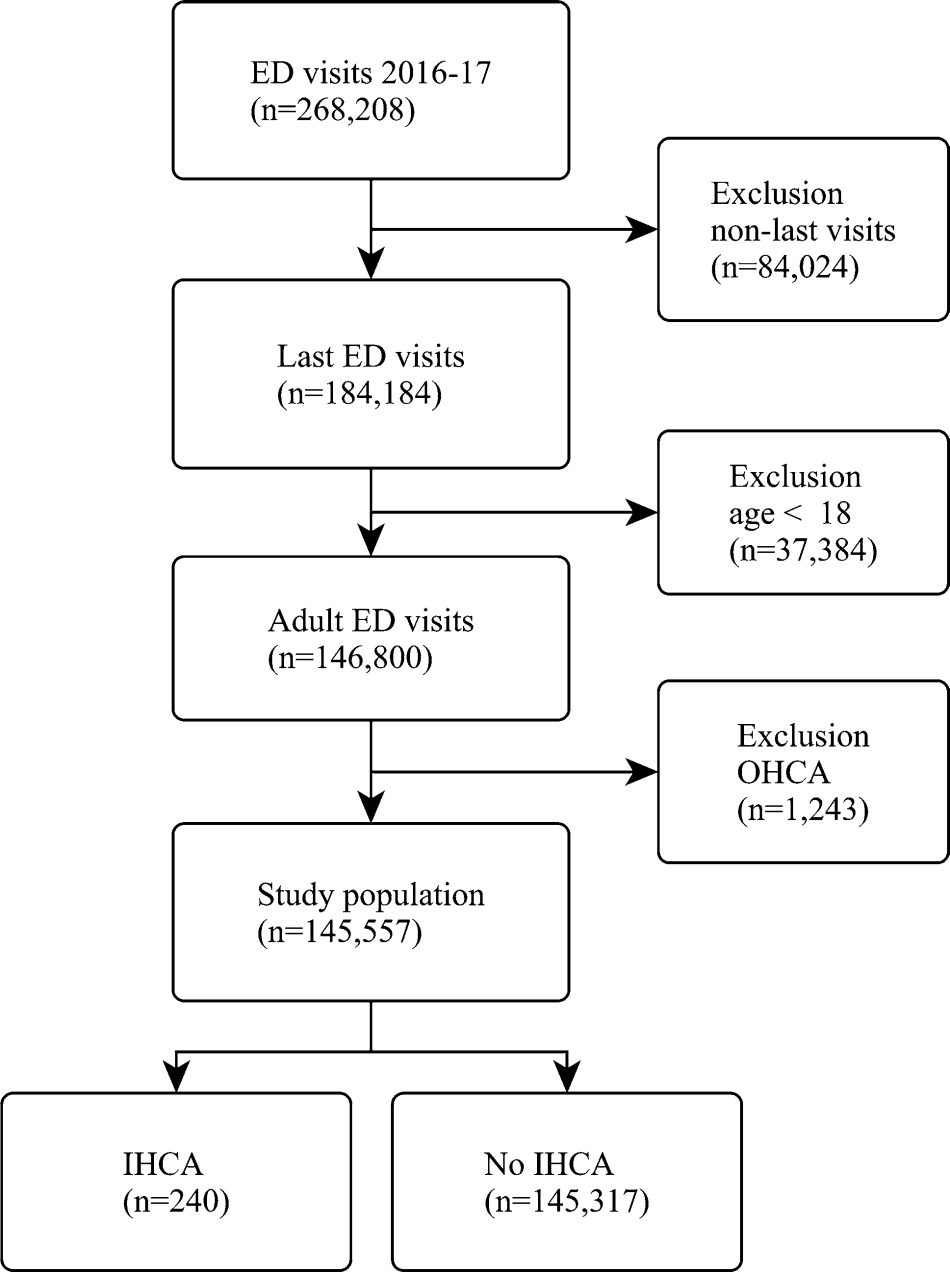
Flow diagram of the patient selection process. *ED* emergency department, *IHCA* in-hospital cardiac arrest.

We conducted a retrospective cohort study using data from the Medical Database of the Far Eastern Memorial Hospital (FEMH), a tertiary academic medical center with approximately 1,200 beds and 130,000 ED visits per year. This database serves as a central clinical data repository for all electronic medical records of the healthcare system, including inpatient, outpatient, and ED records. The electronic database houses various information of the patient, including demographics, diagnosis, treatment, imaging, laboratory, prescription, nursing, billing, and administrative data. In this investigation, we retrieved data from 268,208 ED visits in the FEMH from January 1 2016 to December 31 2017. For those with multiple visits, we selected the last visit per patient to maximize the statistical power for the cardiac arrest analysis. Cardiac arrest may result in death during an ED visit, and that visit became the last visit for the patient. Besides, patients younger than 18 years of age or those who presented with out-of-hospital cardiac arrest (OHCA) were excluded. The subject selection process is shown in Fig. 1. ED-based IHCA was defined as the cardiac arrest that occurred in the ED. This study is a retrospective study of medical records and all experimental protocols have been approved by Institutional Review Board (IRB) of FEMH (IRB number: 108026-E) and was conducted in compliance with the Declaration of Helsinki in 1964. The informed consent was waived by the IRB of FEMH.

### Data collection

The patient’s demographics and time-stamped clinical information at triage were retrieved, including chief complaint on presentation, mode of arrival, transfer status, vital signs (temperature, heart rate, systolic and diastolic blood pressure, respiratory rate, oxygen saturation), and levels of consciousness coded as Glasgow Coma Scale (GCS). The data extractors were hospital information technology engineers who were blinded to the study hypothesis. After the investigators’ meetings, the data underwent electronic cleaning, and invalid data were designated as missing values. Pain scores were evaluated on a numeric rating scale (NRS) of 0 to 10, where 0 indicated no pain and 10 indicated the worst pain imaginable. We further categorized the NRS scores into no (0), mild (1–3), moderate (4–6), and severe (7–10) pain^19^. We also classified levels of consciousness as severe coma (GCS ≤ 8), moderate coma (9–12), and minor coma to normal status (GCS ≥ 13)^20^. Emergency department shifts were classified as day (07:00–14:59), evening (15:00–22:59), and night (23:00–06:59).

We retrieved the five-level computerized Taiwan triage and acuity scale (TTAS), which contains information on 179 structured chief complaints. The chief complaints included OHCA, which was used to identify and exclude the patients experiencing OHCA. The TTAS classifies patients based on computerized algorithms: level 1, resuscitation; level 2, emergent; level 3, urgent; level 4, less urgent; and level 5, non-urgent. This acuity scale has been validated against hospitalization and length of ED stay^21^.

### Outcome measure

The primary outcome measure, ED-based IHCA, was identified via a cardiopulmonary resuscitation (CPR) code (i.e., treated cardiac arrest). ED-based IHCA was defined as the cardiac arrest that occurred while patients were still in the ED. Patients with do-not-resuscitate (DNR) status were not included in the treated cardiac arrest. According to consensus guidelines on reporting IHCA^22^, the incidence of ED-based IHCA was calculated as the number of treated arrests (numerator) divided by the ED study population (denominator).

### Statistical analysis

Summary statistics are presented as proportions [with 95% confidence intervals (CIs)], means [with standard deviations (SDs)], or medians [with interquartile ranges (IQRs)]. Bivariate associations were examined using Student’s t-test, the Mann–Whitney test, and chi-squared test, as appropriate. We used complete-case analysis as the vast majority of variables in the analysis had few or no missing values.

The EDICAS, an 8-item composite score with a possible range of 0 to 13, included age, arrival mode, and categorized vital signs with simple cutoff points (supplemental eTable 1). We fit the eight variables comprising the EDICAS in a multivariable logistic regression model to examine the independent association between each variable and the ED-based IHCA. We also calculated each patient’s EDICAS and computed its sensitivity and specificity with varying cutoff points. The discriminatory ability of the EDICAS was evaluated by using the area under the receiver operating curve (AUROC). For comparison purposes, we also computed the AUROCs by using other EWSs, including the Modified Early Warning Score (MEWS)^12^ and Rapid Emergency Medicine Score (REMS)^23^. Finally, the test characteristics of the EDICAS were computed using varying cutoff points. We conducted two sensitivity analyses to test the robustness of our findings. First, we excluded patients with DNR status (n = 293) and repeated the AUROC analysis. Second, we tested the EDICAS performance separately in non-traumatic and trauma patients.

All odds ratios (ORs) and beta-coefficients are presented with 95% confidence intervals (CIs). All analyses were performed using Stata 16.0 software (StataCorp, College Station, TX). A 3-D figure for visualizing IHCA data was plotted using Python matplotlib v3.2.1. All *P* values are two-sided, and those less than 0.05 were considered to be statistically significant.

## Results

Of the 268,208 ED visits over the 2-year study period, 184,184 unique patient visits were included in our study. After excluding those aged less than 18 years or with out-of-hospital cardiac arrest, 145,557 patient visits served as the study population, including 240 cases of IHCA and 145,317 cases of non-IHCA. The patient selection process is illustrated in Fig. 1. In our study population, the mean age of these patients was 47 years, and 52% were women. The overall incidence of ED-based IHCA was 0.16% (95% CI 0.14 to 0.19%). As shown in Table 1, patients with IHCA were much older, predominantly male, and were likely to present to the ED in the winter in comparison with non-IHCA patients. In terms of weekend or time of ED presentation, there were no significant differences between these two groups. Compared with non-IHCA patients, IHCA patients were more likely to arrive by ambulance, to be transferred from other facilities, and to present with dyspnea and chest pain. IHCA patients were less likely to present with injuries. IHCA patients were more likely to be categorized to higher triage levels, with altered mental status, but were less likely to express pain of any levels. Regarding vital signs at triage, IHCA patients were more likely to present with lower body temperature, lower heart and higher respiratory rates, and lower systolic blood pressure and oxygen saturation status. The median length of ED stay was about 4 h in the IHCA group, and was about 2 h in the non-IHCA group. Compared with non-IHCA patients, the admission and ED mortality rates were significantly higher among patients with IHCA.

**Table 1:** Baseline clinical characteristics of emergency department patients by in-hospital cardiac arrest status.

Variable	IHCA (n = 240)	No IHCA (n = 145,317)	P value
Age, mean (SD), yr	64.4 (18.5)	47.3 (19.1)	< 0.001
Female sex, n (%)	99 (41.2)	74,953 (51.6)	0.001
**Season, n (%)**			0.002
Spring (Mar.–May)	65 (27.1)	35,201 (24.2)	
Summer (Jun.–Aug.)	48 (20.0)	37,972 (26.1)	
Fall (Sep.–Nov.)	52 (21.7)	39,270 (27.0)	
Winter (Dec.–Feb.)	75 (31.2)	32,874 (22.6)	
Weekend, n (%)	83 (34.6)	48,804 (33.6)	0.743
**Time of presentation, n (%)**			0.106
7 am to 3 pm	98 (40.8)	50,715 (34.9)	
3 pm to 11 pm	94 (39.2)	59,080 (40.7)	
11 pm to 7 am	48 (20.0)	35,522 (24.4)	
Arrival by ambulance, n (%)	143 (61.1)	20,318 (14.8)	< 0.001
Transfer in, n (%)	19 (8.1)	5,874 (4.1)	0.002
**Presenting chief complaint, n (%)**			< 0.001
Abdominal pain	12 (5.0)	21,488 (14.7)	
Fever	6 (2.5)	12,773 (8.8)	
Dyspnea	59 (24.6)	5,977 (4.1)	
Dizziness	4 (1.7)	10,170 (7.0)	
Chest pain	18 (7.5)	5,918 (4.1)	
Other	141 (58.8)	88,991 (61.2)	
**Triage level, n (%)**			< 0.001
1	145 (60.4)	3,902 (2.7)	
2	60 (25.0)	17,498 (12.0)	
3	32 (13.3)	90,282 (62.1)	
4	3 (1.3)	30,471 (21.0)	
5	0 (0)	3,164 (2.2)	
Trauma, n (%)	67 (28)	55,837 (38)	0.001
Pain score, n (%)*			< 0.001
Severe (7–10)	8 (3.4)	10,101 (7.0)	
Moderate (4–6)	27 (11.3)	61,963 (42.7)	
Mild (1–3)	3 (1.3)	7,965 (5.5)	
No pain (0)	200 (84.0)	65,231 (44.9)	
GCS < 15, n (%)	125 (52.1)	7,113 (4.9)	< 0.001
Body temperature < 36 °C, n (%)	58 (24.2)	30,320 (20.9)	< 0.001
**Heart rate, mean (SD), beats per minute**			< 0.001
< 60 beats per min	20 (8.3)	3,289 (2.3)	
60–90 (reference)	135 (56.3)	77,717 (53.5)	
> 90 beats per min	85 (35.4)	64,311 (44.3)	
Respiratory rate ≥ 22 breaths per min, n (%)	65 (27.1)	8,194 (5.6)	< 0.001
Oxygen saturation < 95%, n (%)	38 (15.9)	3,912 (2.7)	< 0.001
Systolic blood pressure < 90 mmHg, n (%)	19 (7.9)	1,523 (1.1)	< 0.001
Length of ED stay, median (IQR), hr	4.3 (2.2–10.9)	1.8 (0.9–4.3)	< 0.001
**Discharge status, n (%)**			< 0.001
Discharge	0 (0)	111,512 (76.8)	
Admission	127 (52.9)	25,530 (17.6)	
Death	100 (41.2)	293 (0.2)	
Other	13 (5.4)	7,911 (5.4)	

*IHCA* in-hospital cardiac arrest, *SD* standard deviation, *GCS* Glasgow coma scale, *IQR* interquartile range, *ED* emergency department, *ICU* intensive care unit.

*Available in 145,498 patients.

**Figure 2:**
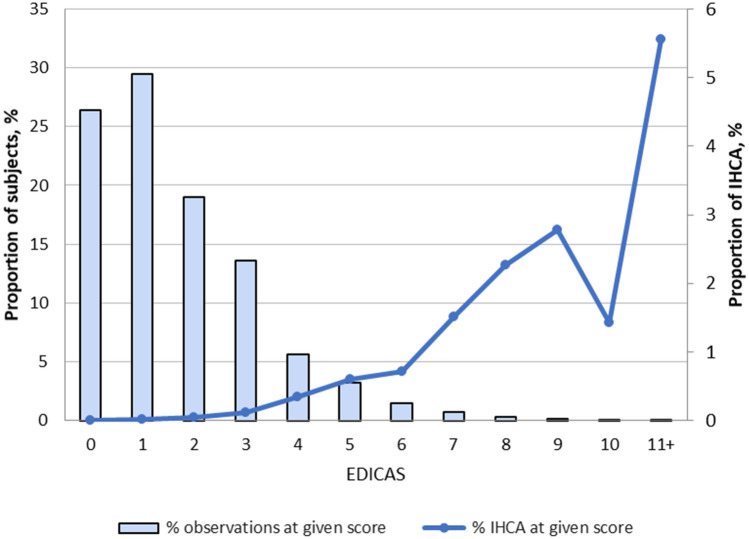
The distribution of EDICAS and proportion of IHCA in each EDICA category. *EDICAS* Emergency Department In-hospital Cardiac Arrest Score, *IHCA* in-hospital cardiac arrest.

Multivariable analysis showed that all components in the EDICAS remained statistically significant, including older age, arrival by ambulance, low systolic blood pressure (< 90 mmHg), brady- (< 60/min) and tachycardia (> 90/min), low oxygen saturation (< 95%), tachypnea (≥ 22/min), hypothermia (< 36 °C), and altered mental status (GCS < 15) (Table 2). The distribution of the EDICAS was shown in Fig. 2 with the proportion of IHCA in each EDICAS category.

**Table 2:** Multivariable analysis of in-hospital cardiac arrest using emergency department in-hospital cardiac arrest score (EDICAS) as predictors.

Variable	Adjusted odds ratio	95% confidence interval	P value
Age ≥ 65 years	2.89	1.99–4.20	< 0.001
Arrival by ambulance	3.41	2.41–4.83	< 0.001
Systolic blood pressure < 90 mmHg	2.66	1.48–4.77	0.001
**Heart rate**			
< 60 beats per min	3.68	2.06–6.55	< 0.001
60–90 (reference)	1.00		
> 90 beats per min	1.59	1.09–2.31	0.016
Oxygen saturation < 95%	2.75	1.79–4.21	< 0.001
Respiratory rate ≥ 22 breaths per min	3.72	2.52 – 5.49	< 0.001
Body temperature < 36 °C	1.81	1.27–2.60	0.001
GCS < 15	2.23	1.49–3.33	< 0.001

*GCS* Glasgow coma scale.

**Figure 3:**
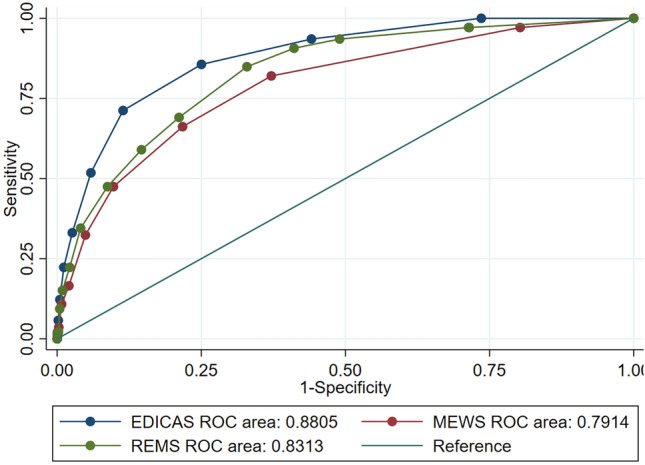
Receiver operating characteristic curves for three early warning scores: EDICAS, MEWS, and REMS. The diagonal line represents a model of no discriminatory ability. *EDICAS* Emergency Department In-hospital Cardiac Arrest Score, *MEWS* Modified Early Warning Score, *REMS* Rapid Emergency Medicine Score.

**Figure 4:**
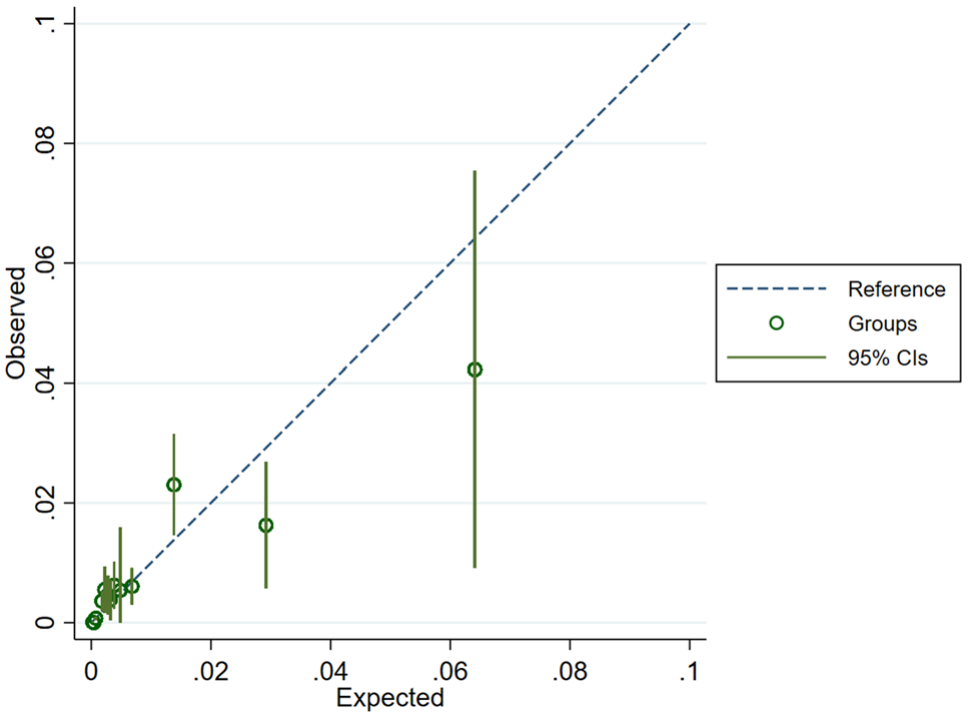
A calibration plot showed the agreement between the observed and predicted probability of IHCA. *IHCA* in-hospital cardiac arrest.

The EDICAS, including age, arrival mode, and categorized vital signs with simple cutoffs, showed excellent discrimination with an AUROC of 0.88 when applied to the ED population (Fig. 3). The AUROC of the EDICAS outperformed those of other EWSs (0.80 for Modified Early Warning Score [MEWS] and 0.83 for Rapid Emergency Medicine Score [REMS]) in the same ED population (p < 0.001 for MEWS; p = 0.002 for REMS vs. EDICAS). A calibration plot (Fig. 4) showed a good agreement between the observed and predicted probability of IHCA within approximately 1% risk. A fair agreement was noted at a higher-risk range (> 1%) of IHCA.

The test characteristics of the EDICAS are shown in Table 3. An EDICAS of 6 or above (i.e., high-risk patients) corresponded to a sensitivity of 33%, a specificity of 97%, a positive likelihood ratio of 12.2, and overall accuracy of 97%.

**Table 3:** Test characteristics of the emergency department in-hospital cardiac arrest score (EDICAS).

Cutpoint	Risk category	Sensitivity, %	Specificity, %	PPV, %	NPV, %	LR +	LR−	Correctly classified, %
≥ 1	Low	100	26	0.1	99.99	1.4	0.0	27
≥ 2		94	56	0.2	99.98	2.1	0.1	56
≥ 3	Medium	85	75	0.4	99.97	3.4	0.2	75
≥ 4		70	89	0.6	99.95	6.1	0.3	88
≥ 5		51	94	0.9	99.93	8.7	0.5	94
≥ 6	High	33	97	1.3	99.92	12.2	0.7	97
≥ 7		22	98	1.9	99.91	18.8	0.9	99
≥ 8		12	99	2.5	99.90	24.6	0.9	99
≥ 9		6	99	2.8	99.90	27.5	0.9	99
≥ 10		2	99	2.7	99.90	27.0	0.9	99
≥ 11		1	99	5.1	99.90	49.9	0.9	99
≥ 12		0	100	0	99.90	0	1	99

*PPV* positive predictive value, *NPV* negative predictive value, *LR +* positive likelihood ratio, *LR−* negative likelihood ratio.

In the sensitivity analyses, when we excluded the DNR patients, the AUROC did not materially change (0.88). Similarly, when we tested the EDICAS performance in medical and trauma patients, and its AUROC did not materially change (0.87 in medical and 0.88 in traumatic patients).

## Discussion

In this ED-based study of 145,557 patients, we externally validated a triage tool, the EDICAS, for predicting imminent IHCA in the ED. Overall, this simple 8-item triage tool outperformed other previously established EWSs and remained to possess excellent discriminatory power in another ED population with a different case-mix. The logical next step would be to embed the EDICAS in the EMR system with appropriate interventions to evaluate its real-world impact in the ED.

Recent epidemiological evidence has suggested that the characteristics of ED-based IHCAs are quite different from those of ward-based IHCA^1,5^. Previous studies revealed that the median time to cardiac arrest was approximately two days in ward patients^2,23^. However, patients in the ED could develop IHCAs within hours after ED arrival. Such a short interval from ED arrival to cardiac arrest in these patients was commonly associated with the time-sensitive nature of certain emergent situations, such as acute coronary syndrome, acute respiratory distress, and so on. Indeed, chest pain and dyspnea were the most common presenting complaints in our IHCA population, a finding that is consistent with the original study. Hence, how to promptly recognize and appropriately manage these high-risk patients within hours is an important issue in the hectic ED environment. We externally validated the EDICAS in a different ED population, and its excellent performance was sustained, suggesting its clinical utility as a screening measure at ED triage for imminent IHCAs in the ED^11,12,24^.

In the current study, our estimate of IHCA incidence was about 1.6 per 1,000 ED visits, which was similar to that in the original study (1.9 per 1,000 visits). In addition to the similar prevalence of IHCA, the test characteristics and the model performance were also remarkably similar in the current study. For example, an EDICAS of 6 or above was used to define the high-risk group in the original study, corresponding to a specificity of 98% and a positive likelihood ratio of 12.7. Using the same cutoff, the high-risk EDICAS corresponded to a specificity of 97% and a positive likelihood ratio of 12.2 in the current study. The AUROC of the EDICAS was 0.86 in the interval validation of the original study, and this figure even raised a bit to 0.88 in the current study. Taken together, these robust results suggested that the EDICAS could be used to raise the possibility of ED-based IHCA and to prompt the second level of physician review. Moreover, the EDICAS outperformed the general EWSs (MEWS and REMS)^25,26^, supporting its role as an ED-specific predictive tool for ED-based IHCA. Alternatively, the EDICAS can be used particularly in patients already triaged to levels 1 or 2, further raising its positive predictive value of IHCA.

There were several potential limitations in the study. First, as this external validation was done in another tertiary medical center, a prospective multicenter study would be ideal to further validate the EDICAS in different hospital settings. For example, differences in triage methods, ED length of stay, and emergency medical services systems exist between hospitals. Second, the real-world clinical impact was not addressed in the current study. The EDICAS may be integrated with the EMR system as clinical decision support with sending alerts to ED staff. It would be of interest to see whether there would be an increase in lead time to identify and intervene high-risk patients, thereby potentially preventing ED-based IHCA and associated deaths. Finally, as our study was retrospective in nature, the results were susceptible to selection and information bias.

In summary, we externally validated EDICAS, a novel 8-item ED triage tool predicting IHCA with the excellent discriminatory ability and test characteristics. We also demonstrated the superior performance of EDICAS over other pre-existing EWSs. The logical next step would be to embed the EDICAS in the EMR system with appropriate interventions to evaluate its real-world impact in the ED.

## Supplementary Information

Supplementary Information.
