# Supplementary material for: External validation of a triage tool for predicting cardiac arrest in the emergency department
Source: Sci Rep. 2022 May 24;12:8779. doi: 10.1038/s41598-022-12781-6 (PMC9130149; doi:10.1038/s41598-022-12781-6)
Supplement: Supplementary file 1 — Supplementary Information. [file 41598_2022_12781_MOESM1_ESM.docx]

**Online Supplemental eTable 1.** The items and scoring of Emergency Department In-hospital Cardiac Arrest Score (EDICAS). The 8-item score ranges from 0 to 13.

| **Variable** | **Scoring** | | |  |
| --- | --- | --- | --- | --- |
|  | **1** | **2** | **3** | |
| Age, year |  | ≥ 65 |  | |
| Arrival by ambulance | Yes |  |  | |
| Systolic blood pressure, mmHg |  |  | <90 | |
| Heart rate, beats per min | <60 or > 90 |  |  | |
| Body temperature, °C |  | <36 |  | |
| Respiratory rate  , breaths per min |  | ≥ 22 |  | |
| Oxygen saturation, % | <95 |  |  | |
| GCS < 15 or acute change in levels of consciousness | Yes |  |  | |

Abbreviations: GCS = Glasgow coma scale.
